# LEMS: a language for expressing complex biological models in concise and hierarchical form and its use in underpinning NeuroML 2

**DOI:** 10.3389/fninf.2014.00079

**Published:** 2014-09-25

**Authors:** Robert C. Cannon, Padraig Gleeson, Sharon Crook, Gautham Ganapathy, Boris Marin, Eugenio Piasini, R. Angus Silver

**Affiliations:** ^1^Textensor LimitedEdinburgh, UK; ^2^Department of Neuroscience, Physiology and Physiology, University College LondonLondon, UK; ^3^School of Mathematical and Statistical Sciences and School of Life Sciences, Arizona State UniversityTempe, AZ, USA; ^4^CAPES Foundation, Ministry of Education of BrazilBrasilia, Brazil

**Keywords:** model description language, standardization, simulation, spiking neural networks, model sharing

## Abstract

Computational models are increasingly important for studying complex neurophysiological systems. As scientific tools, it is essential that such models can be reproduced and critically evaluated by a range of scientists. However, published models are currently implemented using a diverse set of modeling approaches, simulation tools, and computer languages making them inaccessible and difficult to reproduce. Models also typically contain concepts that are tightly linked to domain-specific simulators, or depend on knowledge that is described exclusively in text-based documentation. To address these issues we have developed a compact, hierarchical, XML-based language called LEMS (Low Entropy Model Specification), that can define the structure and dynamics of a wide range of biological models in a fully machine readable format. We describe how LEMS underpins the latest version of NeuroML and show that this framework can define models of ion channels, synapses, neurons and networks. Unit handling, often a source of error when reusing models, is built into the core of the language by specifying physical quantities in models in terms of the base dimensions. We show how LEMS, together with the open source Java and Python based libraries we have developed, facilitates the generation of scripts for multiple neuronal simulators and provides a route for simulator free code generation. We establish that LEMS can be used to define models from systems biology and map them to neuroscience-domain specific simulators, enabling models to be shared between these traditionally separate disciplines. LEMS and NeuroML 2 provide a new, comprehensive framework for defining computational models of neuronal and other biological systems in a machine readable format, making them more reproducible and increasing the transparency and accessibility of their underlying structure and properties.

## 1. Introduction

Computational models are essential tools for understanding complex systems with many interacting entities. In biology, models have been used to explore the properties of biochemical interactions within cells at the protein and genetic levels (Kitano, 2002; Chen et al., 2004; Feist et al., 2008), as well as to investigate electrical signaling in neurons and networks (Segev and London, 2000; Vogels and Abbott, 2005; Herz et al., 2006). Biologically accurate models can incorporate the dynamical properties of different processes spanning multiple spatial and temporal scales and this approach has recently been used to simulate the complete life cycle of a single bacterium (Karr et al., 2012). In neuroscience, multi-scale modeling is particularly important for understanding how low level non-linear mechanisms underlie brain function. For example, models have shown how ion channels present on the membrane of neurons affect higher level properties including neuronal computation (Poirazi et al., 2003; Rothman et al., 2009; Farinella et al., 2014), network excitability (Vervaeke et al., 2012) and neuronal network dynamics (Traub et al., 2005; Vervaeke et al., 2010; Marder and Taylor, 2011). The value of models built from well characterized low level experimental measurements is that they can test the physical plausibility of hypotheses and make quantitative predictions about higher level properties, that can then be tested experimentally. Detailed multi-scale models also provide a mechanism to consolidate and refine knowledge about the properties of brain regions which are increasingly being gathered and organized in great detail (Thomson and Lamy, 2007; Bezaire and Soltesz, 2013). However, for computational modeling to be more widely adopted as a scientific tool in neuroscience, it is crucial that models are made available in accessible formats that allow them to be easily reproduced, compared and critically evaluated by a wider range of scientists.

Models in neuroscience and systems biology are increasingly being made available through repositories including ModelDB (Hines et al., 2004), the BioModels database (Le Novère et al., 2006) and the CellML Model Repository (Lloyd et al., 2008). In neuroscience, most models of the electrical behavior of neurons and networks are built and made available in the specialized scripting languages of domain specific simulators that have been used to construct the model. This is problematic for model and component exchange, accessibility and reproducibility because the structure of the code is simulator and programmer specific, making it difficult to understand exactly how model components were implemented and even to reproduce the data in a published paper. Moreover, porting of models to different platforms is extremely time consuming making cross-simulator validation impracticable in many cases (Gleeson et al., 2010). An additional complication is that researchers working at different levels of description and in different fields implement models using a diverse set of approaches, simulation tools, and computer languages resulting in fragmentation in model specification and implementation.

The need to make computational models more Reproducible, Accessible, Portable, and Transparent (RAPT) has led to the development of a number of simulator-independent model specification languages in computational biology. Two different strategies have been adopted: domain-specific and generic approaches. The Systems Biology Markup Language, SBML (Hucka et al., 2003), focuses on allowing existing simulation tools to share machine readable representations of biological processes using a domain-specific approach. The primary goal is to capture the commonalities of the internal representation of the biological systems modeled in different simulators. The language contains a variety of biological concepts that are widely implemented in systems biology simulation tools such as reactants, products and well stirred compartments. A related approach has been used for NeuroML version 1.x (Gleeson et al., 2010), which has been developed for representing neuroscience models and contains concepts from this field such as ion channels, synapses and neuronal morphologies. This, together with the hierarchical structure of such domain-specific languages, makes the model descriptions compact and easy to understand for users who are familiar with the field. This “building-block” approach has enabled developers to add support for models expressed in NeuroML to a wide range of applications^1^. However, within this framework the data and logic required to fully describe and execute the model is spread across the model scripts, the documentation of the model description language (e.g., Supplementary Text 1 describing NeuroML v1.x in Gleeson et al., 2010) and the simulation engine (e.g., NEURON Carnevale and Hines, 2006 and GENESIS Bower and Beeman, 1997). This hampers the exchange of models between software tools and their transformation into human readable formats, limiting the RAPT of models defined in such formats. In contrast to domain-specific languages, generic model description languages such as CellML (Lloyd et al., 2004) provide a lower level description of the mathematical expression of a model. This provides an unambiguous mathematical representation of the model without requiring any additional domain-specific knowledge. This approach provides machine readability and considerable flexibility for implementing new mechanisms as they are discovered, without the need to alter the inner structure of the language. However, the lack of intrinsic structure within such generic approaches has the disadvantage that models can be represented and constructed in many different ways, making it harder to work with and combine models from different sources. This flexibility also makes generic languages harder to implement and makes verifying an application's compliance to the language specification more difficult.

Here we present a new machine readable declarative language for describing complex physio-chemical systems, that is sufficiently flexible to support new domain specific concepts, yet allows models to be defined in a manner with little redundancy. These properties of the Low Entropy Model Specification (LEMS) language arise from its nested hierarchical structure and the fact that the general definition of model components is separated from instantiations of models with particular parameter values. Moreover, the internal variables of models are defined in terms of their dimensions allowing automated consistency checking and facilitating the handling of units. This bottom up approach enables domain specific knowledge of the system to be incorporated in a compact, machine readable representation without resorting to text based specifications, thereby improving the RAPT of models defined in this format. We show how LEMS can be used as a flexible, low level model description language upon which higher level domain specific languages such as NeuroML version 2.0 can be built. We demonstrate that the current version of the LEMS/NeuroML 2 framework is sufficiently complete and advanced to fully specify a range of synaptic, neuronal and network models. Moreover, we establish that LEMS is sufficiently generic to enable model specification across domains as different as computational neuroscience and systems biology (De Schutter, 2008).

## 2. Results

### 2.1. Dimensional quantities

**Figure 1:**
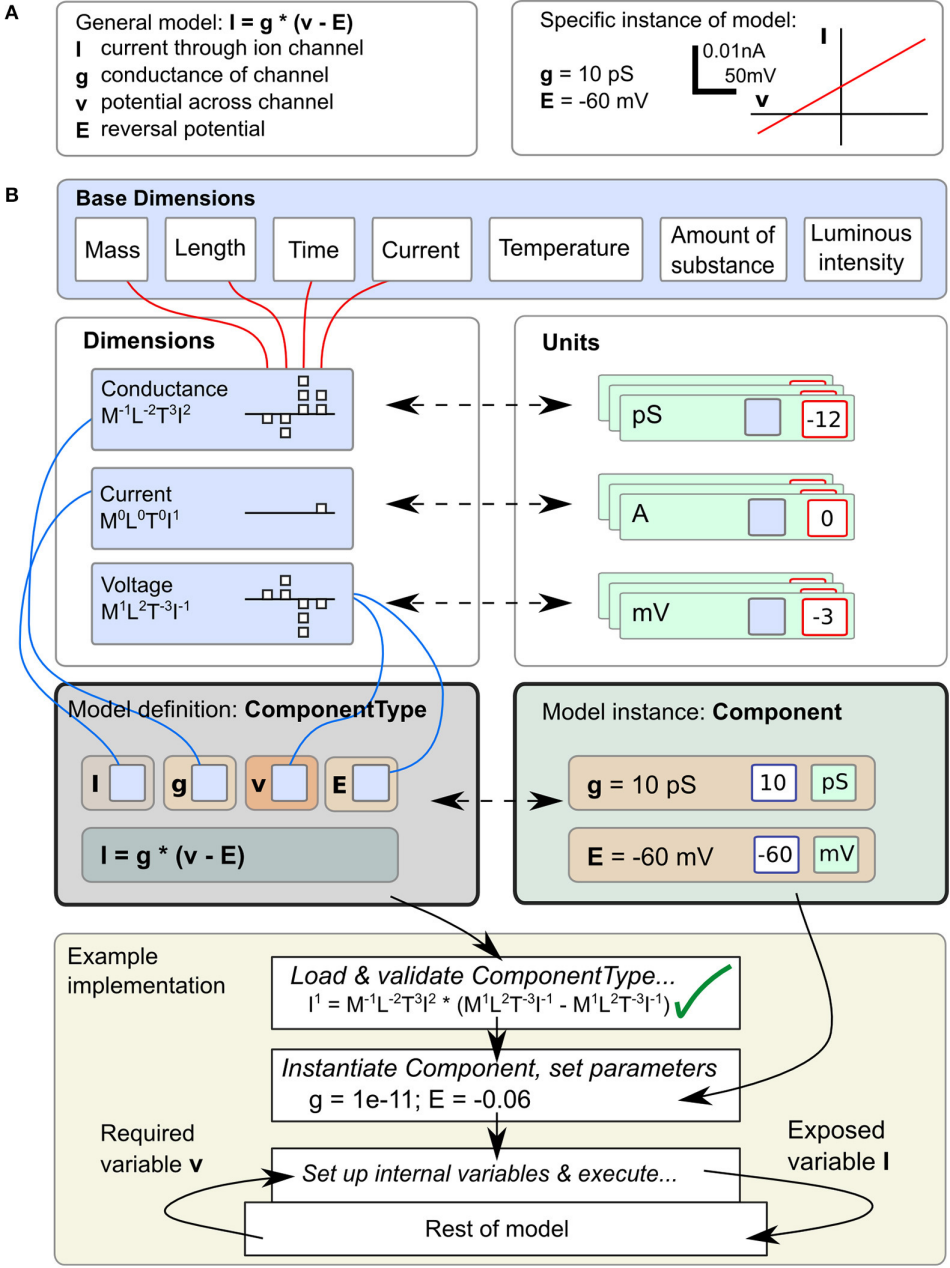
**Definition and use of Units and Dimensions in *ComponentType*s and *Component*s. (A)** A simple model of the current flowing through an ohmic (passive) channel (left), along with a specific instance of the model (right). **(B)** Parameters and state variables are treated as rich dimensional quantities in LEMS. The necessary dimensions are defined in terms of the seven standard SI base dimensions. Units are constructed by combining a reference to a dimension and a scale factor (typically a power of ten). The equations of the model defined in **(A)** are specified inside a *ComponentType* definition. Each parameter or variable must specify its dimensions by reference to one of the dimension elements. A particular instance of a family of models, defined by a *Component* element supplies values for the parameters, consisting of a numerical value and a reference to a unit element (compatible with the specified dimension). The bottom panel shows how the *ComponentType* and *Component* definitions are combined when a model is executed. Typically, a simulator will convert parameters values to a preferred set of units, such as the SI system as shown here, after checking the equations for consistency. In this example, the potential *v* is read from the enclosing scope, the current *I* is computed and exposed for use by other parts of the model.

Correct handling of the dimensions and units of physical quantities is central to LEMS. An equation such as *I* = *g* · (*v* − *E*), for the current *I* through an ion channel of conductance *g*, where the membrane potential is *v* and the reversal potential for the permeant ion is *E* (Figure 1A, left), is as much a statement about the dimensions of current, conductance and voltage as it is about their magnitudes in a particular context. However, when this equation is converted to computational form with fixed values for parameters *g* and *E* (Figure 1A, right) a simulator will typically end up operating on pure numbers. At some stage the dimensions and units must be stripped off. There are broadly three ways this can be done. First, the simulator could require the user to do it, just taking dimensionless quantities for *g* and *E* and expecting the user to interpret the resulting number for *I* correctly. In this case the simulator would function correctly with any consistent set of units, but all the work must be done by the user. Second, the simulator could require the user to add units to the equation, effectively expressing one instance of the general case such as *I*[in mA] = *g*[in Siemens] · (*v*[in mV] − *E* [in mV]). Conceptually *I*, *g*, etc. are still bare numbers but there is some associated metadata carried with them that can be used to check for unit compatibility in assignments. Third, the simulator can represent *I*, *g*, *v*, and *E* as dimensional quantities and handle any transformation into and out of particular unit systems itself without involving the user at all. The shift from the first to the third approach represents a migration of knowledge from the modeler down to the model description language or simulator. LEMS takes the third route in order to conserve knowledge of the system in a machine readable form and to express models in a way that is as close as possible to the modeler's conception of them. The definition and use of dimensional quantities in LEMS is illustrated in Figure 1B. Compound dimensions are defined in terms of the 7 fundamental SI units normally with integer powers (e.g., Area = Length^2^). At this stage named dimensions must be set up for all the quantities occurring in the model. For each dimension, a set of units are defined specifying the power of ten required to scale the unit with respect to its SI equivalent. When a class of models is defined, each quantity just needs a reference to the appropriate dimension. When an instance of the model is specified, a modeler sets the values of the free parameters by giving a numerical value and choosing one of the units compatible with the parameter's dimension. It is worth noting that this approach is nothing other than standard dimensional analysis which is implicit whenever physical models are mathematically expressed. The novelty is in making it part of the formal model specification system rather than requiring modelers to convert their models to dimensionless quantities or to a standard set of units before writing them down.

### 2.2. Separating equations from parameter values: components and componenttypes

In the biological literature, parameter values are often embedded in equations. For example, the expression of a particular ohmic current like the one in Figure 1 may contain an explicit value for *E*, the reversal potential of the charge carrying ion for the solutions in question. This makes it inflexible and hard to study the dependencies of models on changes that would vary those values while preserving the overall structure. To avoid this problem, LEMS enforces a clean separation between the form of the model or family of models and the parameter values that define a particular member of the family. Model families are expressed by defining *ComponentType*s which specify the parameters a particular type of model can contain and the references to other models it requires. A particular member of the family is then defined by creating a *Component* which contains a reference to the corresponding *ComponentType* and provides values for the parameters and references. In Figure 1B, the equation defining the current is in the *ComponentType*, while the parameters that define the instance of the model correspond to the *Component*. As well as defining the parameter types, a *ComponentType* definition can also contain specifications for the dynamics of the model, such as differential equations governing the time evolution of state variables and functions defining new quantities in terms of variables and parameters.

This separation of equations from parameter values typically results in a *three* layered structure for a LEMS model. Firstly, there is a small set of *ComponentType* definitions containing parametrized equations but no actual values. Secondly, there will be one or more *Component* definitions that sets the structure and values for a particular instance of a *ComponentType*. Finally, the actual model that is run may consist of multiple instances of the *Component*s defined in the model. For example, the LEMS specification of a network of integrate and fire neurons could contain just one specification of the basic spiking neuron *ComponentType*, a few definitions of neuron *Component*s that vary in their parameters for particular classes of neurons, and a great many actual neurons. Each cell would have a number of unique state variables, but would refer back to its *Component* definition for the fixed parameter values, and to the single *ComponentType* definitions for the dynamics. In the example in Figure 1, the first two layers are illustrated, but the passive channel instance could be used in multiple places in the rest of the model. These properties of LEMS enable large-scale models with many units to be defined compactly, without losing the flexibility to define heterogeneity in the behavior across units.

### 2.3. Model specification

**Figure 2:**
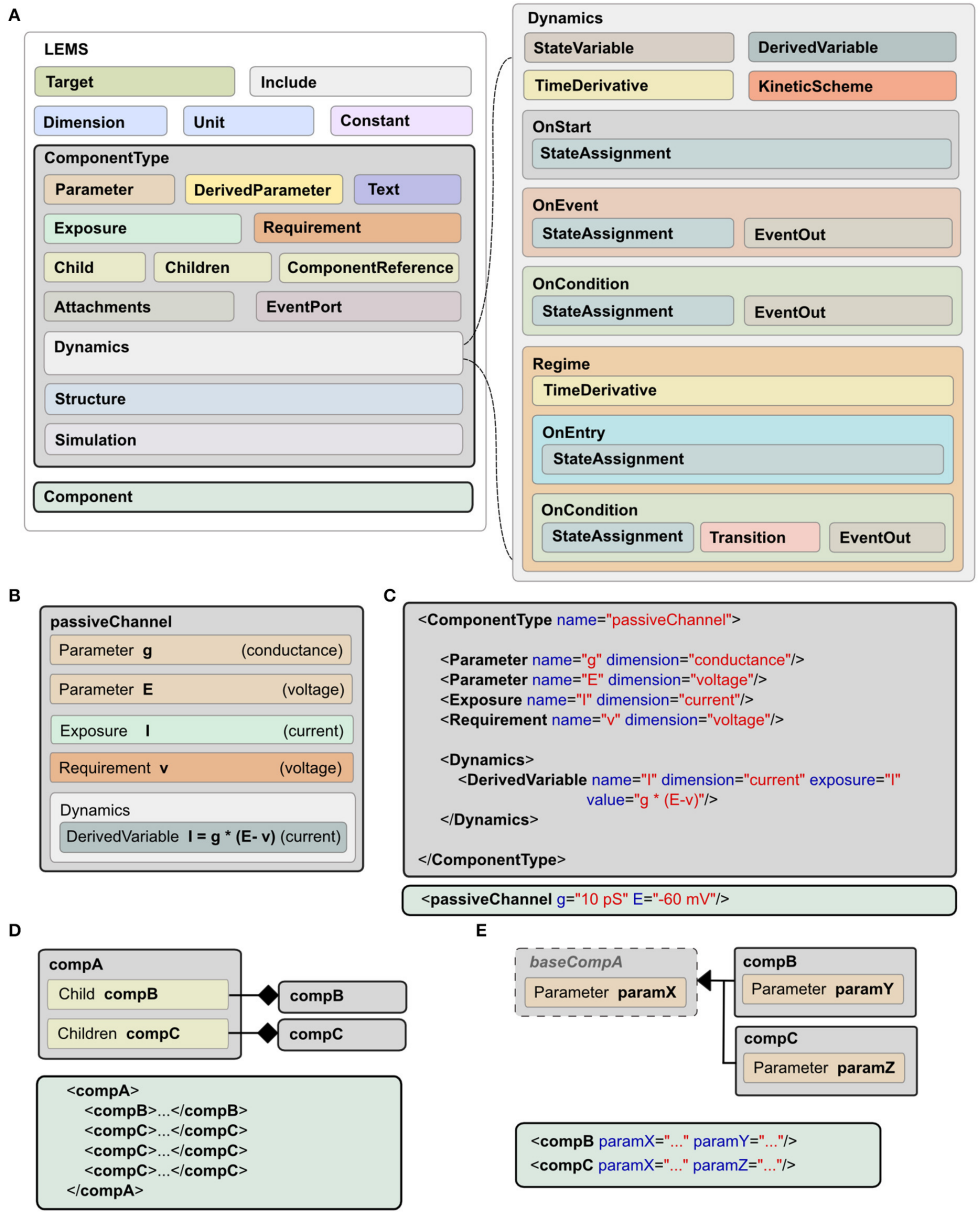
**Structure of LEMS models. (A)** Models in LEMS are specified using *ComponentType* definitions with nested *Dynamics* elements. Any *Parameter* or *StateVariable* declaration must refer to a *Dimension* element defined at the top level. A *Component* element sets parameter values for a particular instance of a *ComponentType*. Each *Parameter* value must refer to one of the *Unit* elements defined at the top level. The *Dynamics* element supports continuous time systems defined in terms of first order differential equations, and event driven processing as specified by the various “*On…*” elements. Multiple *Regime*s, each with independent *TimeDerivative* expressions can be defined, along with the rules to transition between them. **(B)** Example of a *ComponentType*, the passive channel model from Figure 1. **(C)** The XML equivalent of the *ComponentType* (top) and *Component* (bottom) for this model. **(D)** Defining containment in LEMS, using *Child* (exactly one sub element of the given type) or *Children* (zero or multiple copies). **(E)** Extension in LEMS. Extending *ComponentType*s inherit the structure of the base type. Example *Component*s in XML are shown in **(D,E)**.

A model expressed in LEMS takes the form of a tree of elements in which each element can only contain children of particular types. The root element, *Lems*, can contain seven types of child elements as illustrated in Figure 2A: *Target*, *Include*, *Dimension*, *Unit*, *Constant*, *ComponentType*, and *Component*. The *Target* element points to the main *Component* in the model, i.e., the one to be simulated. The *Include* element is for including LEMS definitions from other files. A *Dimension* element associates a name with powers for each of the seven SI base dimensions as illustrated at the top of Figure 1B. A *Unit* element associates a symbol with a dimension, a power of ten, and optionally a scale and offset for non-metric units such as Fahrenheit. The *Constant* element is provided for expressing physical constants such as the elementary electric charge or the gas constant.

As described earlier, a *ComponentType* specifies the structure and dynamics of a class of models that share the same underlying mathematical description. A particular set of *Parameter* values corresponding to a single instance of a model based on this general type is expressed as a *Component*. An example is shown in Figure 1, where the *ComponentType* (middle, left) defines the generic passive channel model, and the *Component* (middle, right) specifies a particular instance. In addition, a *ComponentType* says what types of child elements are allowed or required in corresponding components. Full details of the *ComponentType* definition can be found in the online language specification^2^. The key elements are illustrated in Figure 2A. They are:

- *Parameter*: a quantity, defined by name and dimension, that will need to be set in the corresponding *Component* definition.
- *DerivedParameter*: a quantity that is a function of the *Component*'s *Parameter*s, which does not change with time.
- *Text*: a text string used for labeling and referencing expressions (e.g., the name of the ion species associated with a channel).
- *Exposure*: a quantity that is accessible to other elements, such as the membrane potential of a cell which can be used by its children.
- *Requirement*: a quantity that must be accessible in the containing hierarchy as an *Exposure* on an ancestor element.
- *Children*: specifies a type of child element which a *Component* is allowed to contain. Zero or more children of this type can be present in an instantiated *Component*.
- *Child*: an individual, named child element. In contrast to *Children*, one and only one of this *Component* must be present.
- *ComponentReference*: a reference to a *Component* of a particular type elsewhere in the model. This allows the same *Component* to be used from different places (e.g., a reference to a parameterized ion channel within a channel density specification).
- *Attachment*: operates like the *Children* element but for dynamically created children. This can be used, for example, for synapses that are only added to a target cell when there is a network connection to it.
- *EventPort*: for sending and receiving discrete events.

The elements described so far cover the structure of a *Component* involving parameter values, references to other components and nested child components. They do not say anything about how a *Component* behaves. A key feature of LEMS is to define, in a declarative format, how hybrid models evolve with time. This is achieved with the *Dynamics* element as shown on the right of Figure 2A. Each *StateVariable* or *DerivedVariable* refers to a *Dimension* element to give the dimensions of the variable. First order differential equations for how the *StateVariable*s change with time are expressed with the *TimeDerivative* element. The *StateAssignment* element coupled with *OnStart* or *OnEvent* allow for discrete changes in *StateVariable*s, on initialization or on the arrival of an event respectively. The expressions for the values of the *TimeDerivative* and *DerivedVariable*s are functions of the *StateVariable*s and *Parameter*s. The grammar for mathematical expressions is close to that used in the C programming language except that boolean relations involving the <and> symbols are replaced by their Fortran equivalents (.lt.,.gt. etc) to avoid XML encoding problems.

In addition to sets of differential equations, *Dynamics* elements also support nested *Regime* elements and *KineticScheme* elements. *Regimes* allow the dynamics of a *ComponentType* to be expressed via a finite state machine. Each regime has its internal dynamics, and conditions on which transitions between regimes occur are specified using the *OnCondition* element. The *KineticScheme* supports the specification of systems that can be in one of a small number of states at any time with probabilistic transitions between states. In particular, this includes continuous-time Markov processes as are used for stochastic models of ion channels.

The *Structure* element specifies how a *Component* should be interpreted when a simulator constructs an executable instance of a model. By default, each *Component* in a model gives rise to a single instance of its state variables when the model is executed. The state variables are then governed by the dynamics definition in the associated *ComponentType*. Elements in the *Structure* declaration can be used to change this behavior, for example to make multiple instances of the state variables, or to instantiate a different component. A typical application for the latter would be a *Component* that defines a population of cells. The population *Component* might define the number of cells it contains but would refer to a *Component* defined elsewhere for the actual cell model to use.

In addition to the components described above for specifying the structure, behavior and parameter values of models, LEMS also supports a *Simulation* element, which includes descriptions of the essential quantities for defining how a model should be run and what should be recorded or plotted. This details the model, the timestep, simulation time, and the outputs that should be stored to files or displayed. This is not intended to be a comprehensive simulation specification language, but rather it allows a single LEMS file to contain all the information required to set up a model, run a standard simulation, and record the results. This has helped considerably with specifying tests and validating models across simulators. It also facilitates sharing models. For example, executable LEMS files containing *Simulation* elements are available for each of the models shown in the figures in this paper. There is an automated mapping from this simplified format for simulation parameters to the more widely used Simulation Experiment Description Markup Language (SED-ML, Waltemath et al., 2011), as outlined in Section 2.10.

### 2.4. Expressing physical connections and containment

A key concept in many biological models is that one entity is “part of” another. In LEMS this is expressed by the hierarchical nesting of elements within *Component* definitions (Figure 2D). For example, in a Hodgkin and Huxley type neuronal model, an ionic conductance can have one or more sets of two-state gates (Hodgkin and Huxley, 1952). The gates open or close to control the flow of ions through the conductance, according to the membrane potential. In the LEMS representation, the gates are child elements of the channel. Likewise, if a given gate possesses internal dynamics, then this should be expressed as child elements of the gate. This child relationship (or containment) implies that a *Component* can have access to the properties exposed by its enclosing elements higher up the hierarchy. Thus, a voltage dependent gate of a channel associated with a specific cell has access to the membrane potential of that cell but not of any other cell in the network. Hierarchical nesting therefore defines the model structure by setting the allowed relationships between quantities in models. This property of LEMS contrasts with more abstract languages such as VHDL (VHSIC Hardware Description Language, IEEE, 1988) or NineML (Raikov and De Schutter, 2012), which require that components be explicitly connected by ports. Such descriptions are more flexible with the consequence that models must include additional scoping rules to restrict them to physically and biologically plausible configurations. By contrast, LEMS incorporates knowledge of biological and physical relationships into the nested hierarchical structure. This feature enables concise representation of physically consistent models and clearly distinguishes them from physically inconsistent ones.

### 2.5. Conceptual model hierarchy

As well as belonging to families, models are often organized into classes that extend or refine a particular set of attributes. For example, synapses may broadly be defined as *Component*s that are placed on a target cell, receive discrete events that can occur with a specified delay, and affect the target cell in some way. Then they may be separated into abstract synapses that deliver a discrete impulse, or more biophysically realistic ones that generate a conductance, or a combination of conductances, on the target cell. Such relationships can be expressed in LEMS by constructing an inheritance hierarchy among *ComponentType*s (Figure 2E). A base synapse type can declare that it receives events, listens for a membrane potential on a parent *Component*, and produces a current. Types that extend this base synapse type can add parameters and other attributes to express different subtypes of the model. As well as providing a mechanism for expressing the relationships between families of models, this also provides many of the same benefits as inheritance in class hierarchies in object oriented software. In particular, a *ComponentType* may require children of a particular base type, without needing to distinguish between the many possible extensions. The flexibility of this approach is illustrated in Section 2.8.1 below, which includes a gate on an ion channel model with forward and reverse transition rates, but is indifferent to the functional form that is used.

### 2.6. Model encoding

LEMS has been developed so that XML documents are used to encode models. This choice is driven more by convenience and pragmatism than the desire to develop an XML format *per se*. There are many well-developed technologies for working with XML documents, particularly XPath and XSLT, which prove very useful for operating on LEMS models. There are also libraries in most programming languages to facilitate reading and writing valid XML. However, the main reason for using XML for LEMS is its ability to encode a hierarchical object model rather than the more advanced features of XML. Although XML provides mechanisms for giving elements unique IDs, including files from other sources, and making references between elements, none of these features are used because the document-centric semantics of XML does not match the model-centric scoping rules in LEMS. Rather, LEMS layers its own semantics on top of the core XML concepts of elements, attributes and nesting. This means that it is straightforward to develop alternative encodings for LEMS and map them to the XML format.

### 2.7. Implementations of LEMS

LEMS is supported directly by two newly developed, open source simulation tools: jLEMS written in Java, and PyLEMS (Vella et al., 2014) written in Python. Each of these supports parsing LEMS model definitions, checking dimension and unit consistency, and simulating models. The jLEMS implementation supports a number of approaches for simulating models where the dynamics are defined in terms of ordinary differential equations (ODEs). The default is to evaluate expressions by traversing the parsed *Component* hierarchy and to update state variables with a simple forward Euler numerical scheme operating on the fully expanded component tree. Better performance is achieved by flattening the *Component* tree (i.e., removing children and adding scoped parameters, variables and ODEs for these at the top level) so that tightly coupled quantities are grouped together within the same *Component*s and then solved using a 4th order Runge Kutta scheme. For this to work, it is essential that each group of tightly coupled variables is handled together. This is addressed with another attribute on the *ComponentType* definitions that specifies which ones are suitable for flattening. It is the responsibility of the *ComponentType* developer to set this correctly. For example, if a *Component* only interacts with other *Component*s by delayed events then it is a good candidate for flattening. If it is a child of another *Component* with continuous access to inherited variables then it cannot be flattened on its own.

PyLEMS allows parsing of LEMS models and simulation of their behaviors using a basic forward Euler numerical scheme. It is slightly less comprehensive than the Java implementation; in particular the *KineticScheme* element is not supported. Both of these packages are intended as reference implementations for the LEMS language rather than efficient simulators in themselves. The two libraries form the basis for modules allowing LEMS to be exported to multiple other formats (see Section 2.10), which allows for faster simulation of models on dedicated simulation platforms. PyLEMS and jLEMS can also be used as libraries for adding native support for LEMS to other applications. For example, jLEMS is used as a library in neuroConstruct (Gleeson et al., 2007).

More details on jLEMS and PyLEMS, including how to obtain the latest version of the applications, can be found in the Materials and Methods Section.

### 2.8. Examples of models implemented in LEMS

The following examples illustrate the current scope of LEMS by showing how a range of models that are commonly used in computational biology can be expressed in the language. As described in the Materials and Methods section, source code for all of these examples is available allowing execution on both jLEMS and PyLEMS.

#### 2.8.1. Hodgkin and Huxley type ionic conductances

**Figure 3:**
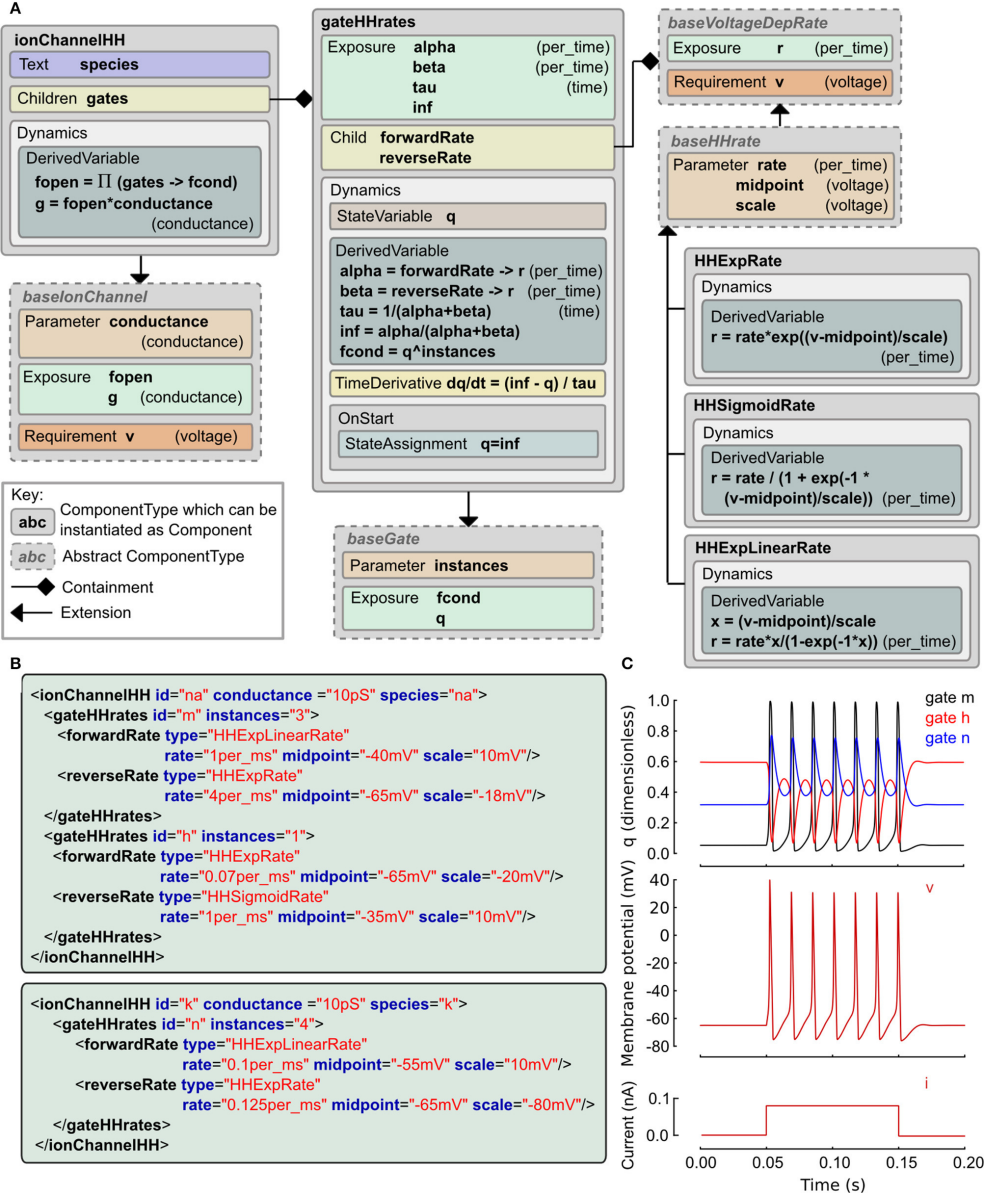
**Example of a Hodgkin Huxley type ionic conductance expressed in LEMS. (A)** Gray boxes indicate *ComponentType* definitions with the connectors expressing containment and extension relationships. The main *ComponentType*, *ionChannelHH*, permits multiple *gateHHrates Component*s as children. The total conductance through the channel is based on the product of fractional conductances through these. The *gateHHrates ComponentType*s calculate forward and reverse rates from child *Component*s which extend *baseVoltageDepRate*. **(B)** *Component* instances that express the standard HH sodium and potassium conductance models. The hierarchical relationships defined among the *ComponentType*s are implemented as nesting of the elements in the *Component* XML with the channel element containing one or two gate elements and each gate containing named transition rate elements for the forward and reverse transitions. **(C)** Behavior of a cell containing these two conductances (together with a leak conductance) in response to a current clamp input. Plots show m, h, n gating variable q (top), membrane potential (middle) and injected current (bottom).

The Hodgkin and Huxley (HH) equations (Hodgkin and Huxley, 1952) have spawned a long lineage of models of active membrane conductances, all using roughly the same equation structure and forms of expressions but with occasional deliberate or accidental changes. The LEMS *ComponentType* definitions for the family of HH type models are shown in Figure 3 along with the XML for the classic HH sodium and potassium channels expressed as LEMS *Component*s. Note that this type of ion channel model could potentially be specified in other, more compact sets of LEMS *ComponentType*s. The containment and inheritance used in this description are influenced by the need for a set of extensible *ComponentType*s for use in NeuroML 2, as outlined in Section 2.9.

We use a probabilistic interpretation of the HH formalism which reduces to the conventional HH model in the continuous limit. In this version the m and h gating variables are replaced by gating particles and the α and β quantities occurring in the ODEs are replaced by forward and reverse transition probabilities. It is interesting to note that although the published form is purely expressed in ODEs, the original HH paper alluded to this scheme as a possible but, at the time, experimentally unjustified mechanistic interpretation of their equations. In this interpretation, an ionic conductance has one or more gating complexes which consist of one or more instances of a two state gate. The gate can be either closed or open, and the transition between the two states is governed by independent forward and reverse transition rates which may depend on the membrane potential, *v*. Although it is rarely apparent from the way models are published, almost all models use functional forms for these rates drawn from the following three expressions: *f*(*x*) = *e^x^*, *g*(*x*) = 1/(1 + *e*^−*x*^), and *h*(*x*) = *x* / (1 − *e*^−*x*^) where *x* ≡ (*v* − *v*_midpoint_)/*v*_scale_ and the the actual rate is the value of *f*, *g* or *h* scaled by a constant factor.

These relations are captured in the *ComponentType* definitions in Figure 3A. The *IonChannelHH* element extends the *baseIonChannel* element which requires a membrane potential *v* and computes a conductance *g*. The HH specific element has a set of children, each of which is a *gateHHrates*. These contain a *Dynamics* element which computes the behavior of a single gating particle. For this, they require the forward and reverse transition rates between the closed and open states. These are provided by named child elements that extend the *baseVoltageDepRate*. Three extensions to this type are defined covering the three standard functional forms. With these *ComponentType* definitions in place, the majority of HH style models currently in use can be expressed just by defining new *Component* elements. XML for the classic HH sodium and potassium channel models is shown in Figure 3B and the behavior of the state variables for these channels when placed on a cell is shown in Figure 3C. A complete specification of the model in LEMS including the definition of the dynamical behavior of the cell and input current is included in the zip file of the Supplementary Material.

#### 2.8.2. Adaptive exponential integrate and fire neuron model

**Figure 4:**
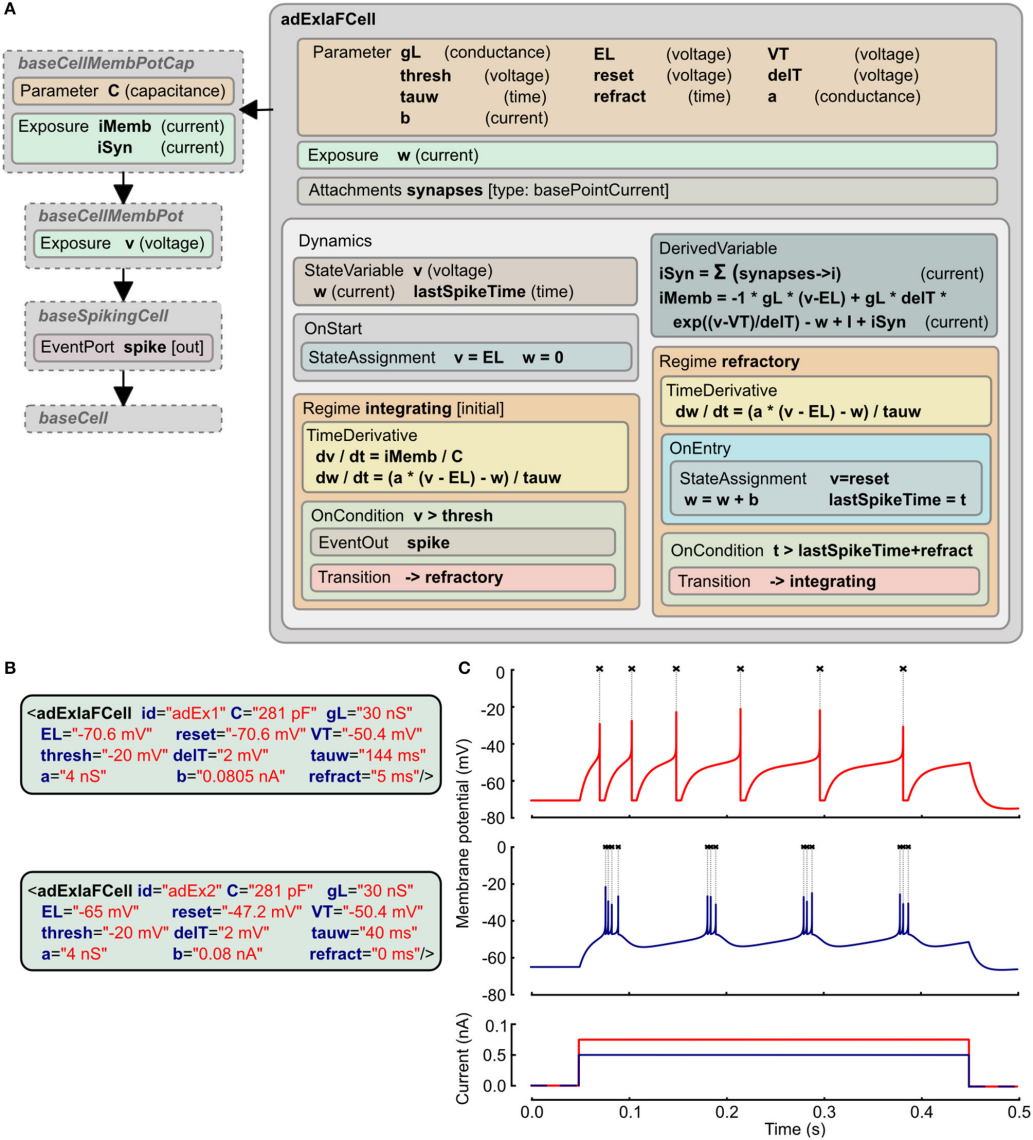
**Example of Adaptive Exponential Integrate and Fire neuron model expressed in LEMS. (A)** The *ComponentType* definition of the model defines the *Parameter*s required for the model and the two *Regime*s, with their distinct behaviors and the conditions for transitions between them. The *adExIaFCell* is an extension of more generic types, from *baseCell* indicating it is part of a broad class of “cells,” *baseSpikingCell* indicating that it is a cell that emits spiking events, and *baseCellMembPot* which specifies an exposed variable *v* for the membrane potential with dimension voltage. *baseCellMembPotCap* adds that the cell will have a dimensional capacitance *C*, and exposes identified current components across the membrane and due to synaptic input. **(B)** Examples of *Component*s specifying particular instances of Adaptive Exponential Integrate and Fire neurons. **(C)** Traces showing spiking behavior of the two cells from **(B)** due to current injected at 50 ms (0.75 nA top, 0.5 nA bottom). Crosses mark points where spiking event is emitted.

The adaptive exponential integrate and fire model has two state variables, one for the membrane potential (*v*) and the other (*w*) for an adaptation current that changes according to the spiking activity of the cell (Brette and Gerstner, 2005). The LEMS *ComponentType* definition of this is shown in Figure 4A. It makes use of two distinct *Regime*s in the *Dynamics*: one for normal integrating behavior, and one for the refractory phase. The *OnEntry* element in the refractory regime applies the membrane potential reset and the discrete change in the adaptation current. The exit condition for this regime is that the refractory period due to the last spike is complete.

The XML for two instances of this neuron model is shown in Figure 4B. Since the *ComponentType* definition does not declare any *Child* or *Children* elements, the XML has no nested elements and simply contains a set of parameter assignments for each of the parameters declared in the *ComponentType*. Traces of the membrane potential from each of these model instances when brief current pulses are applied are shown in Figure 4C.

#### 2.8.3. Simulation of a synapse with short-term plasticity and non-linear postsynaptic conductance

**Figure 5:**
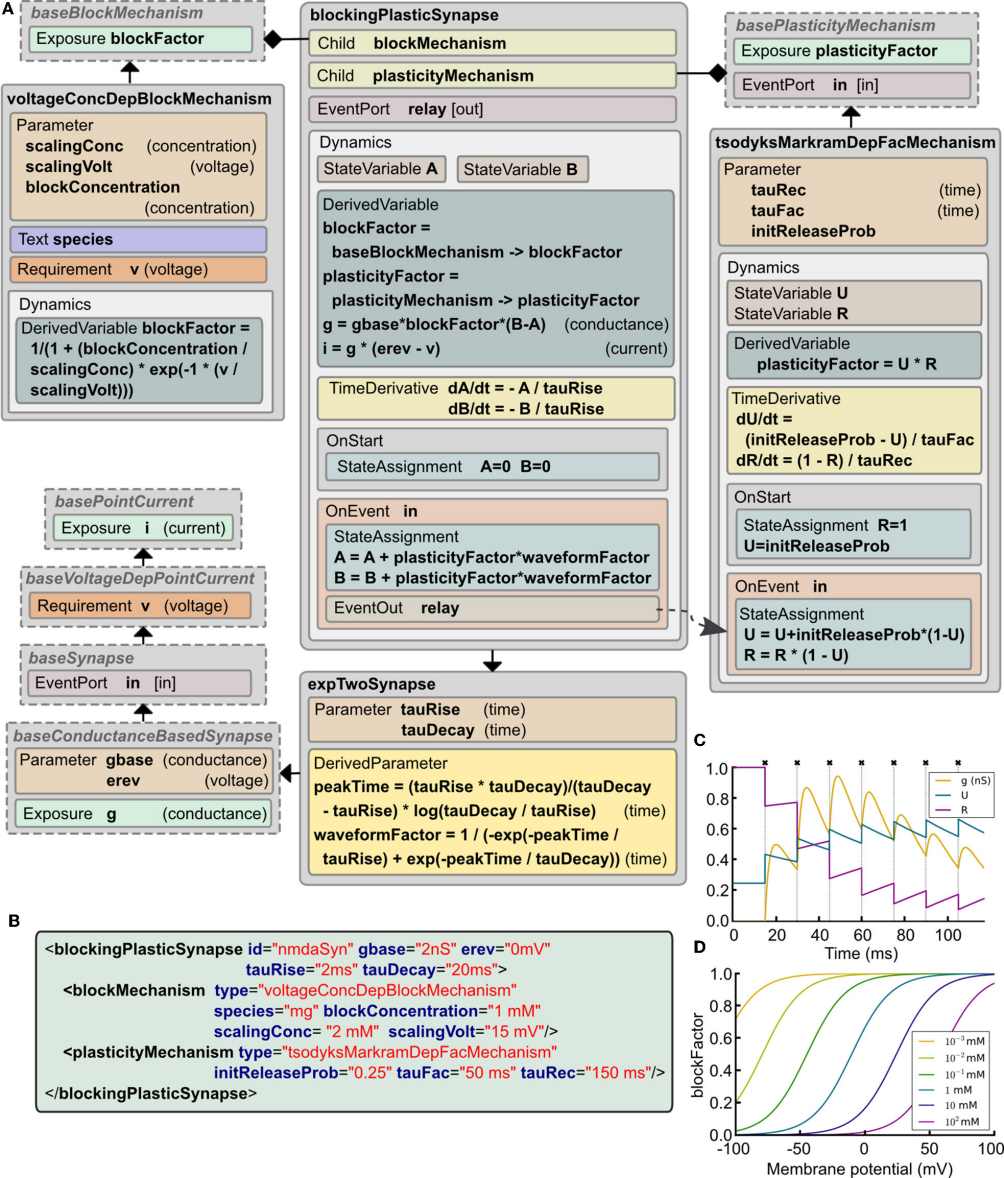
**Example of a complex synapse model expressed in LEMS. (A)** The *Dynamics* block in *blockingPlasticSynapse* specifies the evolution of the synaptic conductance *g* and how the synaptic current *i* depends on it and on the postsynaptic membrane potential *v*. In this example, a *tsodyksMarkramDepFacMechanism ComponentType* provides a short term plasticity model based on Tsodyks et al. (1998), and a *voltageConcDepBlockMechanism ComponentType* provides a simple model of magnesium block. Note how spike timing information is relayed from the synapse model to the plasticity mechanism through a parent-to-child *EventConnection* declared in the *Structure* element in *tsodyksMarkramDepFacMechanism*. **(B)** XML code describing an NMDA receptor mediated synapse with plasticity (parameter values chosen to illustrate behavior in **C,D**). **(C)** Behavior of the synaptic conductance *g* and the state variables defining plasticity, *U* and *R*, during synaptic stimulation (crosses show input events), with the postsynaptic cell clamped to 100 mV to ensure complete Mg^2+^ unblock. **(D)** Changes in *blockFactor* with varying membrane potential for different values of Mg^2+^ concentration.

Central synapses often exhibit both presynaptic short-term dynamics and non-linear postsynaptic voltage dependent conductances due to the presence of NMDA-Rs (N-Methyl-D-aspartate receptors). It is possible to implement these synaptic mechanisms in the current version of LEMS. This can be achieved by starting with *basePointCurrent*, which represents anything that generates a current, and gradually adding more properties (Figure 5A). These include: accessing the postsynaptic membrane potential; detecting presynaptic action potentials through an *EventPort*; specifying the parameters for a simple double exponential conductance waveform (baseline conductance, reversal potential and rise and decay times). The *ComponentType blockingPlasticSynapse* defines two child elements corresponding to the voltage-dependent block and short-term plasticity mechanisms. Specific types of these elements are illustrated: *voltageConcDepBlockMechanism* for a widely used model of voltage dependent Mg^2+^ block in NMDA-R synapses and *tsodyksMarkramDepFacMechanism*, for a model of short term plasticity based on Tsodyks et al. (1998). However, new *ComponentType*s based on *baseBlockMechanism* and *basePlasticityMechanism* can be created without requiring any update to *blockingPlasticSynapse*.

Figure 5B shows an example of a synapse model that can exhibit short term plasticity as well as a non-linear voltage-current relationship. Figure 5C shows how the presynaptic state variables and the postsynaptic conductance evolve with time during a high frequency burst of presynaptic action potentials. Figure 5D illustrates the voltage dependence of the blocking factor for different concentrations of Mg^2+^. Thus, a wide range of simple and biologically detailed synaptic mechanisms can be defined in LEMS. Synapses defined in this way can be used in network connections between LEMS *Component*s (Section 2.12) and potentially incorporate delays and scaling factors (weights).

### 2.9. Use of LEMS as basis for type definitions in NeuroML 2

**Figure 6:**
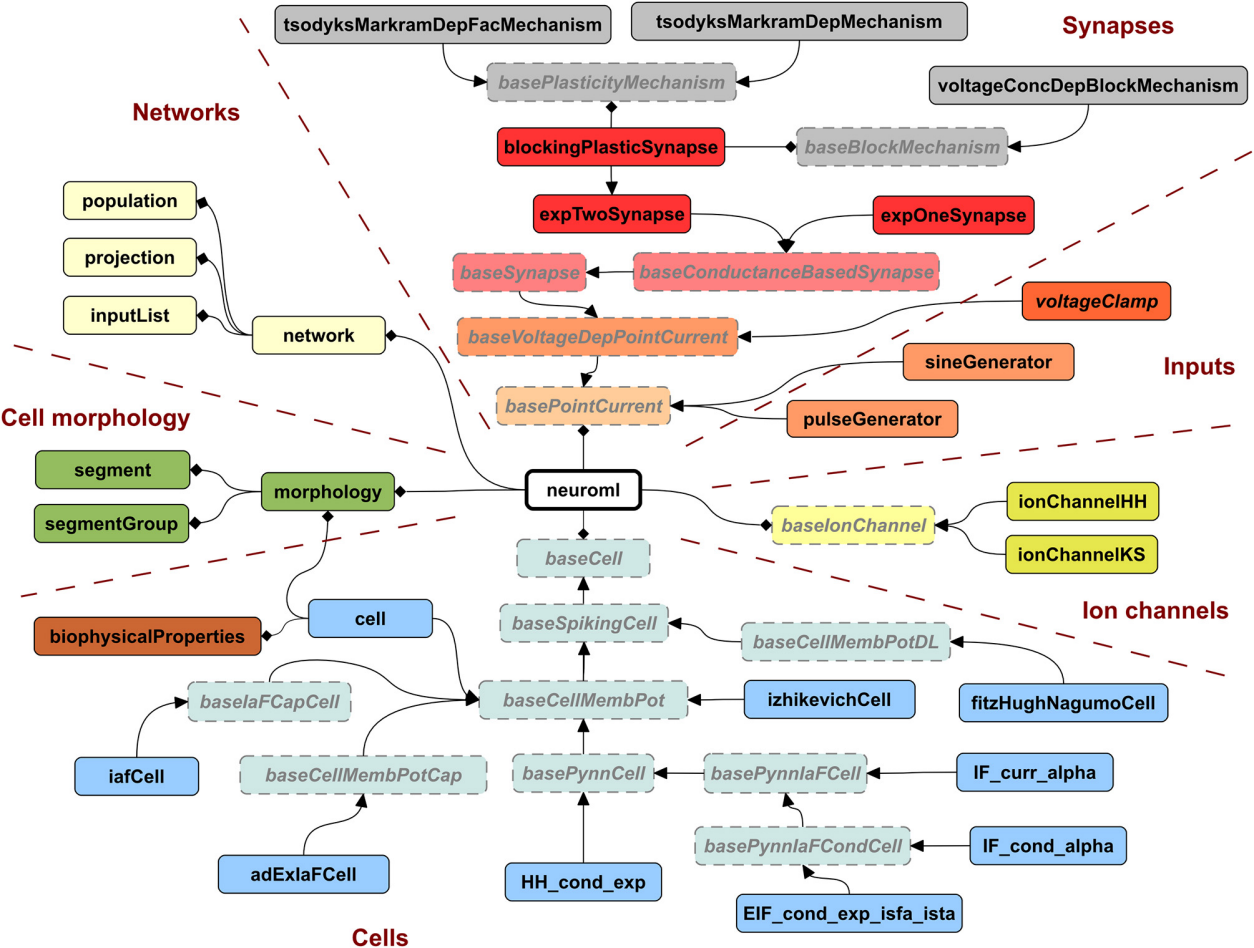
**Hierarchy of ComponentTypes defining the elements of NeuroML 2**. A NeuroML document consists of a root *neuroml* element and subelements of 6 broad classes. All cells extend the *baseCell* type. These include one (*iafCell*) and two (*izhikevichCell*, *adExIaFCell*) variable abstract spiking neuron models, descriptions of the standard PyNN neuron models (extending *basePynnCell*) and the *cell* element (corresponding roughly to the *cell* element of NeuroML v1.x), which contains a description of the *morphology* and the *biophysicalProperties* (passive electrical properties, channel densities, etc.) of the cell. Ion channels can be described by the standard Hodgkin Huxley (*ionChannelHH*, Figure 3) or kinetic scheme (*ionChannelKS*) based formalisms. Inputs to cells produce a time varying current, which could depend on the voltage of the *Component* on which they are placed. Synapses extend the *baseSynapse* type and can have single (*expOneSynapse*) or double (*expTwoSynapse*) exponential conductance waveforms, or have more complex dynamics, as described in Figure 5. Networks in NeuroML consist of lists of *population*, *projection* and *inputList* elements. Morphologies (which can be top level elements or can be children of *cell*) are described by lists of *segment* and *segmentGroup* elements. The elements shown here represent the state of NeuroML 2 at the beta3 release.

A key motivation for developing LEMS was to provide a flexible, low level model description language that was machine readable, thereby overcoming one of the limitations of NeuroML version 1.x. While NeuroML version 1.x can specify the structure of XML elements for ion channels, synapses, and cells, the definitions of their dynamical behavior were only available in text based descriptions (Supplementary information of Gleeson et al., 2010). To address this problem, we now use LEMS *ComponentType* definitions to express the structure and dynamical behavior of elements in NeuroML version 2. Indeed, all of the *ComponentType* elements illustrated in Figures 3–5 are actually part of the NeuroML version 2 specification. Figure 6 provides a more complete view of the *ComponentType* definitions that make up NeuroML version 2, including types which form the basis of broad families of models (e.g., *baseSynapse* and *baseCell*) and those that can be instantiated as components, such as *izhikevichCell*, *voltageClamp* and *expTwoSynapse*. XML files with the complete LEMS specifications for the version of NeuroML 2 used in this paper (version beta3) are included in the Supplementary Material zip file. A full description of the latest version of the NeuroML 2 *ComponentType* definitions is available online at: http://www.neuroml.org/NeuroML2CoreTypes.

An XML file containing NeuroML 2 will only contain *Component* elements based on these standard types and can be validated against an XML Schema document (Materials and Methods), in the same way as a NeuroML version 1.x file. An application that reads NeuroML models can choose either to use LEMS definitions to define their behavior or simply to recognize the NeuroML elements and map each element to its own internal representation. This latter approach loses the extensibility that the generic LEMS definitions affords, but has the advantage that the simulator can use hard-coded and optimized implementations of the standard models.

The *Dynamics* element currently describes the temporal evolution of point process models. The structure of cells with extended morphologies expressed as lists of *segment*s and *segmentGroup*s can be specified in NeuroML 2, and there are corresponding LEMS *ComponentType*s which define their hierarchical relationship (Figure 6), but there is not yet a description in LEMS of how the membrane potential across the cell could be calculated, e.g., by treating the model as a set of connected compartments. This does not prevent a full description of multicompartmental cell model in NeuroML 2 however, and these can still be mapped to simulators which support these models. For example, descriptions of ion channels and synapses can be mapped to NEURON's native format (NMODL, Hines and Carnevale, 2000) using the LEMS descriptions of those model components (see below) and the morphological description in *segment*/*segmentGroup* elements mapped directly to NEURON's internal morphology description format. Native support in LEMS for models that involve scalar fields over three dimensional structures is under development (see Discussion).

The *Structure* element of LEMS underlies the ability of NeuroML to specify *network*s containing *population*s of cells connected with *projections* which pass discrete events through synapse models (e.g., as shown in Figure 5). Due to the declarative nature of NeuroML, it currently only supports descriptions of networks as lists of cell locations and explicit connections, or a small set of network templates for compact network descriptions. More complex network connectivity patterns, potentially with heterogeneous connectivity parameters, can be created either with graphical tools or in a procedural programmatic fashion. An example of the former is neuroConstruct (Gleeson et al., 2007), which can generate complex 3D network models and export them in the explicit list representation of NeuroML 2. An example of the latter is libNeuroML, a Python based API for reading and writing NeuroML 2 (Vella et al., 2014). PyNN is a Python package for simulator independent neural network creation (Davison et al., 2008). Allowing a model specified in PyNN to be exported to NeuroML 2, or to use a model element specified in NeuroML2/LEMS in a PyNN script are scenarios we are actively pursuing to help link these complementary modeling approaches. The ability to express the standard cell types in PyNN as LEMS *ComponentType*s (cells extending *basePynnCell* in Figure 6) is an important step toward this goal.

### 2.10. Interaction of LEMS/NeuroML 2 with other model specification languages and simulators

**Figure 7:**
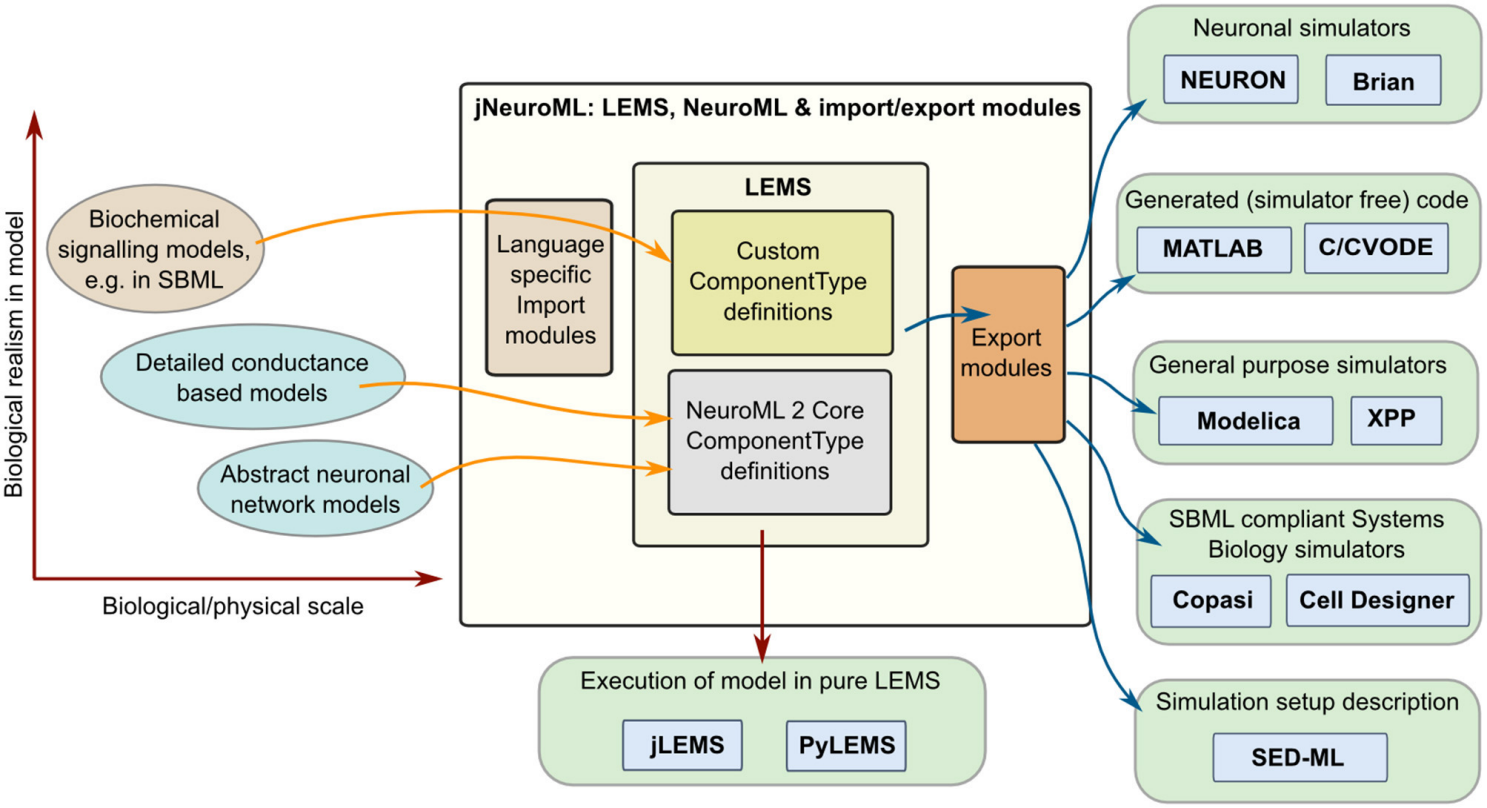
**Interaction of LEMS and NeuroML 2 with other model specification and simulation formats**. Models used in computational neuroscience and systems biology with varying levels of biological detail, and at multiple physical scales can be represented in LEMS, either through the use of predefined, domain specific *ComponentType* definitions (e.g., for NeuroML) or by importing from other structured modeling formats (e.g., SBML). Files in LEMS can then be simulated with the reference implementations, jLEMS or PyLEMS, or exported to a number of other formats. jNeuroML is a collection of utilities in Java for handling these transformations. Export formats include neuronal simulators NEURON and Brian, MATLAB or C code for simulating the models with solvers in these languages, Modelica, XPP and SBML.

Multiple software approaches exist for simulating models in computational biology, from using dedicated simulators in fields such as neuroscience (Brette et al., 2007) and systems biology (Ghosh et al., 2011), to using general purpose packages such as MATLAB, and even generating code (Goodman, 2010) to run an optimized version of the model in low level languages (e.g., C++ or Java). By providing a structured language for model definition LEMS greatly facilitates mapping models to and from these formats. Figure 7 shows some of the options currently available for this. Most of these mapping options are enabled through a Java package for generating multiple formats which builds on jLEMS and an API generated from the NeuroML 2 Schema (Materials and Methods). All of these Java packages, together with the LEMS definitions for NeuroML 2 *ComponentType*s, are bundled into the jNeuroML package^3^, which facilitates accessing this functionality through a command line tool, *jnml*.

Model descriptions from multiple domains can be loaded into an internal representation based on LEMS, either by using predefined domain specific *ComponentType* definitions as in the case of NeuroML, or by mapping existing formats such as SBML to equivalents in LEMS. Once the models are in LEMS they can be simulated with one of the LEMS reference implementations. As mentioned previously, these are not intended to be efficient simulation platforms, and so the internal representation of the model can also be mapped to other formats. Current options (Figure 7) include neuronal simulators NEURON (Carnevale and Hines, 2006) and Brian (Goodman and Brette, 2008); generation of code in MATLAB or C (using, CVODE Cohen and Hindmarsh, 1996) for simulating the model; generating of model scripts in Modelica format^4^; generating XPP (Ermentrout, 2002) scripts; and converting the model to SBML for execution in one of the applications supporting that standard. Initial support for mapping LEMS models to the neuronal model description formats NineML (Raikov and De Schutter, 2012) and SpineML (Richmond et al., 2014) has also recently been added (see Discussion). Note that not all export formats are supported for all NeuroML 2 *Component*s. jNeuroML will throw an error when exporting to a format that does not support a particular model feature, rather than generating incomplete code. The information on simulation duration, timestep, variables to plot and save present in the *Simulation* element of the LEMS file can also be exported to SED-ML format (Waltemath et al., 2011).

### 2.11. Mapping to and from systems biology languages

**Figure 8:**
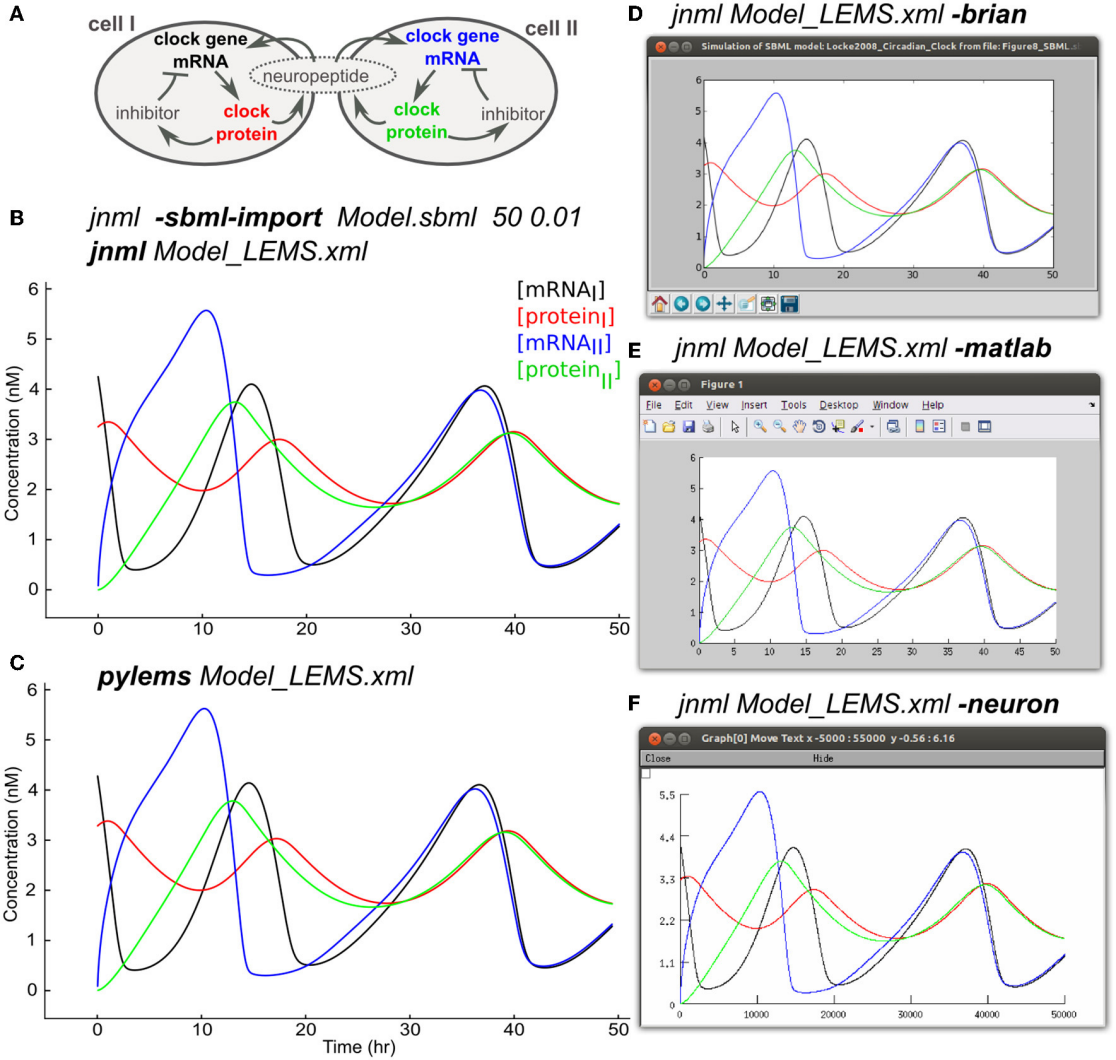
**Importing SBML into LEMS and exporting to multiple simulation formats. (A)** A model of a circadian clock from Locke et al. (2008). Two cells from the suprachiasmatic nucleus contain mRNA for producing a clock protein, which in turn activates a transcriptional inhibitor. A neuropeptide is produced in both cells, the mean level of which affects transcription in each cell, contributing to synchrony in protein synthesis between the cells. **(B)** The model was converted into LEMS format using the jNeuroML utility *jnml* and the option *-sbml-import*, specifying the simulation duration and time step to use for numerical integration. The resultant LEMS file was executed using *jnml* without arguments. The values of all species concentrations and variable parameters are plotted and saved by default in the generated LEMS, but only 4 values are shown here for clarity. **(C)** The LEMS model simulated using PyLEMS, which produced similar results. **(D)** The LEMS model converted to Brian using the *jnml* option *-brian*. A screenshot is shown of the plot produced when the generated Python script is executed. **(E)** The LEMS model converted to Matlab using the *jnml* option *-matlab* and the resultant plot shown. **(F)** The model converted to NEURON using option *-neuron*, and a screenshot is shown of the plot produced after the generated NMODL files are compiled and the simulation script is run. Plots **(C–F)** use the same colors for traces as **(B)**.

Models that contain detailed subcellular signaling pathways are increasingly being used to help understand the complex interactions between ion channels, metabolites and genes within cells (Chen et al., 2004; Feist et al., 2008) and at synapses (Kotaleski and Blackwell, 2010). Many of these models are being made available through resources such as BioModels (Le Novère et al., 2006) and converted to standardized formats such as SBML. The SBML import feature of LEMS allows the majority of SBML files, most of which specify rates of reaction between biochemical species, to be mapped to an equivalent set of ODEs in LEMS. Figure 8A shows an example of this using a model of circadian rhythm generation in the suprachiasmatic nucleus (Locke et al., 2008) which was retrieved in SBML from the BioModels database (BIOMD0000000185), imported into LEMS, and executed with jLEMS (Figure 8B). The LEMS model can also be run directly by PyLEMS (Figure 8C). Use of the command line utility *jnml* facilitates conversion into other formats. The model can be easily converted for execution using Brian, MATLAB and NEURON (Figures 8D–F). Other export options available for this model include CVODE, Modelica and XPP.

Export of LEMS models to SBML is also supported. Due to the monolithic nature of SBML files, this feature is currently only suitable for mapping single *Component*s to SBML. However, the Hierarchical Model Composition package in SBML Level 3 should allow more complex LEMS/NeuroML networks to be mapped to modular SBML models.

The SBML test suite^5^ has been used to test the import feature for SBML. Some of the more advanced features of SBML, such as algebraic rules, delays in events and piecewise expressions in functions are not yet supported, but of those SBML test examples that are supported (813), 78% pass when imported and simulated in jNeuroML.

### 2.12. Representing a simple neuronal network in LEMS and NeuroML 2

**Figure 9:**
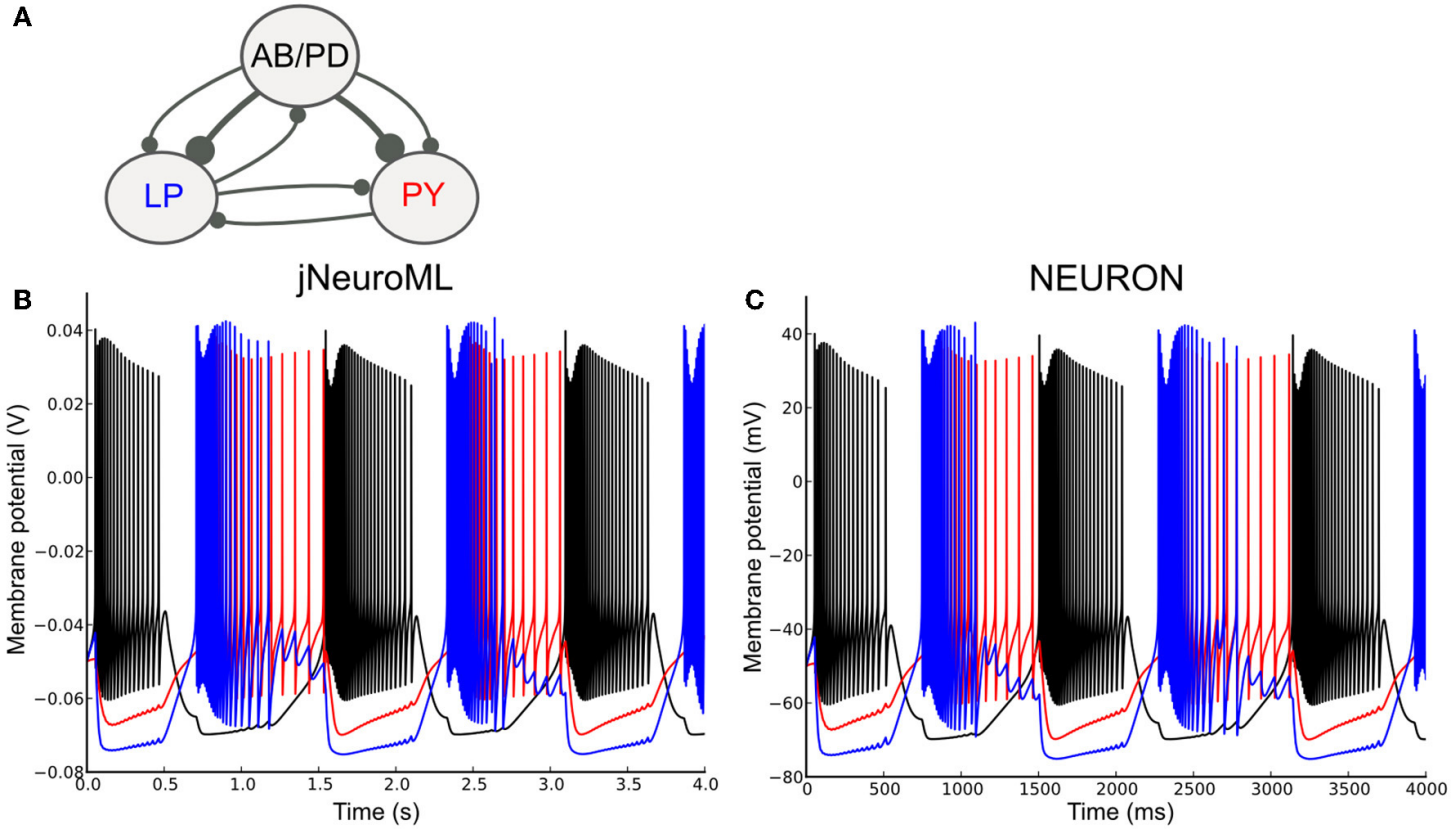
**A network of conductance based cell models specified in NeuroML 2 and simulated using jNeuroML and NEURON. (A)** Model of the pyloric pacemaker network created in NeuroML 2 based on Prinz et al. (2004). This contains a lumped model of the coupled anterior burster and pyloric dilator neurons (AB/PD), a single neuron representing the lateral pyloric neurons (LP) and a single pyloric neuron model (PY). These are connected by inhibitory fast glutamatergic connections (thin lines) and slow cholinergic connections (thick lines). **(B)** The membrane potential for each of the 3 modeled neurons when simulated with jNeuroML. The colors of the traces match those used for the neurons in **(A)**. **(C)** Model converted to NEURON using jNeuroML and executed using this simulator.

To test the ability of LEMS to express networks of complex neurons, we converted a well known network model containing conductance based neurons to NeuroML 2 and used jNeuroML to simulate this. The pyloric network of the crustacean stomatogastric ganglion has been extensively studied as a small network which can exhibit stereotypical rhythmic firing behavior under different sets of neuron and network parameters (Prinz et al., 2004; Marder and Taylor, 2011). Figure 9A shows the simplified form of the network we have converted to NeuroML 2. The three cell models are taken from Prinz et al. (2004), and each includes varying levels of the following currents: a fast Na^+^ current; delayed rectifier and transient K^+^ currents; slow and T-type Ca^2+^ currents; a hyperpolarization-activated inward current; and a [Ca^2+^] dependent K^+^ channel, I_*KCa*_. The Ca^2+^ currents and buffering determine the dynamics of the intracellular Ca^2+^ concentration, which, in turn, controls the gating of I_*KCa*_. The reversal potential for Ca^2+^ is calculated using the Nernst equation (an implementation of the Goldman-Hodgkin-Katz equation for ionic currents is also included in NeuroML 2). The synaptic connectivity is simplified from the original model, using event based chemical synapses as opposed to the graded synapses used in Prinz et al. (2004). A full specification of the channels, cells and network is included in the Supplementary Material zip file.

Figure 9B shows the network model when executed in jNeuroML. The numerical core of this simulator has no inbuilt knowledge of conductances or ion concentrations, and all of the knowledge of the how to handle these diverse, dimensional variables comes from the LEMS definitions of the cells, channels, etc. The LEMS model description can be translated to NEURON's internal format (hoc and NMODL) via the *jnml* utility; and thus can be simulated as any other NEURON model (Figure 9C). All of the dimensional quantities from the LEMS model are converted to a set of consistent units in NEURON (based on mV and ms). The NMODL files contain information on the units for all of the variables that NEURON should use (e.g., conductance and current density) and these have been validated for unit consistency in the generated scripts with the *modlunit* utility in NEURON.

Even though the results for both jNeuroML and NEURON simulations agree qualitatively, membrane potential traces (Figures 9B,C) deviate slightly from each other. Such discrepancies are due to distinct numerical integration schemes used by the simulators, and could be reduced by choosing smaller integration step sizes. Nevertheless, given that some neural systems can be critically sensitive to small perturbations (London et al., 2010), some degree of divergence between numerically calculated trajectories might be expected, given long enough timeseries.

## 3. Discussion

Here we describe LEMS, a flexible model specification language that provides structured, compact and machine readable descriptions of complex models in neuroscience and beyond. By utilizing the 7 base physical dimensions as primitives and forming tree-like nested hierarchical structures, LEMS can specify models in a manner that reflects their underlying physio-chemical properties and structure and provides a framework for automated handling of units. These features, together with the separation of mathematical relationships describing classes of models from the parameters that define a particular model instantiation, provide a flexible framework for building compact, low redundancy (low entropy) model representations. We demonstrate the current functionality of LEMS using Java and Python based implementations of the language and show how it underlies the definition of model components in NeuroML version 2, a standardized format for computational neuroscience, by implementing a variety of different models at the channel, synaptic, neuronal and network levels. Moreover, we also demonstrate that LEMS has a wider application than neuroscience by using it to specify models in systems biology, thereby providing a bridge between these two traditionally separate fields (De Schutter, 2008).

### 3.1. LEMS—a new approach to model specification in neuroscience

**Figure 10:**
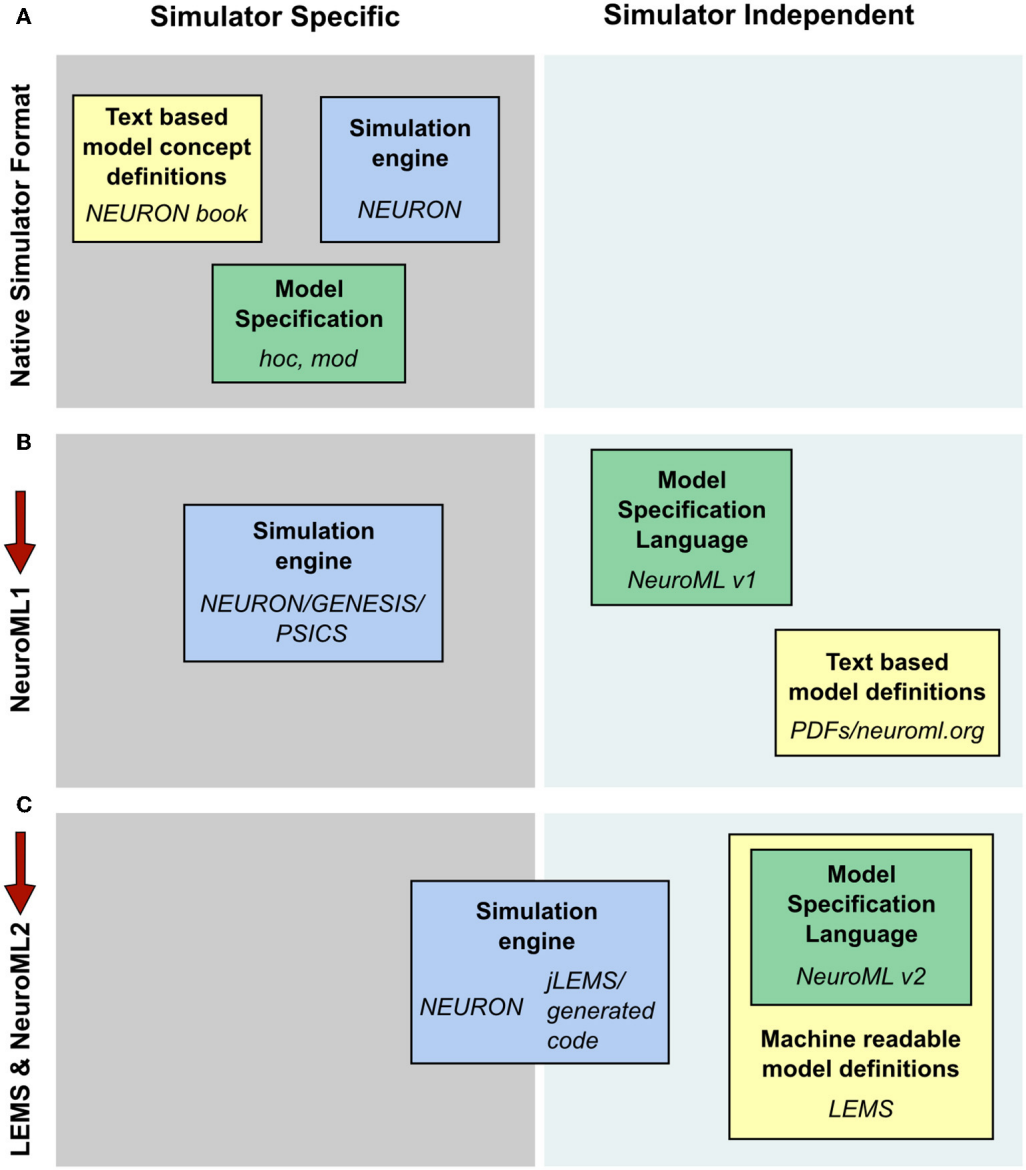
**Segregation of knowledge throughout different modeling approaches. (A)** The complete description (physiological and mathematical) of a simulated model in a simulator like NEURON is generally spread across three locations: the simulation scripts which describe the particular instance of the model (green box, hoc and mod files in the case of NEURON); the description of the concepts underlying the elements used to specify the model, e.g., the membrane and cable equations (yellow box, the NEURON book and online resources); and the code which constitutes the numerical core of the simulator (blue box). **(B)** NeuroML 1 provided a format (green box) to encode the information on model instances as well as resources to describe the model concept behaviors (yellow box), though the latter were still supplied in non-machine readable, text based documentation. Model descriptions could still only be mapped to simulators which had internal implementations of the key neuroscientific concepts being simulated (blue box). **(C)** NeuroML 2 extends the knowledge modalities that can be expressed in machine readable formats, providing a language for describing model instances (green box) while allowing a description of the structure and behavior of key modeling concepts to be specified in LEMS (yellow box). This allows mapping not only to neuroscience specific applications, but also more generic hybrid dynamical system solvers such as jLEMS, or generation of standalone code (blue box). Note that in each case the steps needed to solve equations numerically, such as grid generation and time discretization, are entirely contained within the blue box. The NeuroML descriptions are completely independent of any details of the numerics.

Computational modeling of biological systems typically begins with a physical conception of the entities to be modeled. This is converted into a mathematical representation involving differential equations or stochastic processes that are then solved numerically on a computer as a computational system. Many models in computational neuroscience have traditionally been constructed and simulated using specialized simulators. In this way, the files specifying the model instance, the description of the underlying model types and the implementation of the numerics to solve the model can be quite simulator specific (Figure 10A). The fact that a common set of core model types were used for detailed compartmental modeling by multiple simulators allowed simulator independent specification of model descriptions as well as text based model definitions with NeuroML v1.8.1 (Figure 10B). This scenario still required manual conversion of the model definitions to simulation engine specific formats however, and it was difficult to add new model classes to the framework. These significant limitations spurred the development of LEMS, where a guiding principle in the design has been to migrate knowledge of the modeled system from domain-specific simulators and text based definitions to the model specification language (Figure 10C).

Fully flexible generic computational approaches (e.g., general purpose programming languages such as C), are not well suited for standardizing complex biological models with domain specific concepts. The challenge in developing LEMS was to keep the flexibility of such generic programming approaches while introducing structure within the model specification. This is achieved in LEMS through a bottom up, nested hierarchical structure that is rooted in the base physical dimensions. By having dimensional quantities as the basic primitives of the language, LEMS also eliminates a common frustration in developing models by enabling automated equation validation and unit consistency checking, thereby removing the requirement on the modeler to do these by hand. An equally important feature of LEMS is the separation of model component types from the parameters and their instantiation. This enables the basic properties of a type of models, such as neurons, to be defined only once, multiple variants of this type to be defined and each of these to be used as many times as necessary. This approach enables compact representations of populations of model components, while also providing the flexibility to enable heterogeneity in models to be implemented.

The need to develop and define new model types has long been acknowledged by neuronal simulator developers. NEURON's NMODL format (Hines and Carnevale, 2000) and Brian's simple text based model specification (Goodman and Brette, 2008) cover many of the use cases of developing novel models in a specific simulation environment. The latter even facilitates code generation (Stimberg et al., 2014). Using LEMS to define new model types has the advantage that it is an integral part of a wider framework for model specification, interoperability and exchange, incorporating NeuroML version 2 and tools for reading, writing, simulating and automatically converting the models to multiple simulator formats. The conversion functionality used in this framework is facilitated in large part by the extension mechanisms for individual simulators described above.

Another neuroscience focussed model description language called NineML (Raikov et al., 2011) has been developed in parallel with LEMS and NeuroML 2. NineML has been created by the Task Force on Multiscale Modeling, organized by the International Neuroinformatics Coordinating Facility (INCF). While the overall goals for developing LEMS and NineML are largely similar (and many of the authors of this paper contributed to that Task Force) there are some key differences in the scope and design of the languages. Both languages separate model definition from *Component* instantiation: the NineML “Abstraction Layer” for model specification corresponds roughly to *ComponentType* definition in LEMS, and its “User Layer” contains cell and network creation as present in LEMS *Component*s and NeuroML 2. However, a key difference between these two languages is that NineML does not currently support hierarchical structures or extension among *ComponentType*s and so tends to favor flatter and less structured model representations. Moreover, unlike LEMS, which has physical dimensions as primitives, dimension/unit handling is not currently fully supported in NineML. LEMS also prioritizes succinct representation of the model definitions (e.g., Figures 3B, 4B) whereas NineML is not intended to be human readable. SpineML (Richmond et al., 2014) is a language based on NineML and shares most of these constraints. Another distinction between these approaches is that NeuroML 2 has been designed with a similar overall structure as NeuroML v1.x, to facilitate upgrading of import/export support in applications and mapping of existing model components to the new format. While proliferation of standardized languages can be detrimental, exploring multiple different ways to solve complex problems is beneficial, as long as it results in a more robust end product. To this end we have started to develop features for exporting and importing NineML and SpineML to and from LEMS (through jNeuroML), thereby maximizing the opportunities for sharing and reuse of models specified in these largely complementary languages.

### 3.2. LEMS and systems biology

In systems biology there are several initiatives for providing declarative specification of biological models and simulations, which have underpinned the rapid expansion of this field over the last decade (Ghosh et al., 2011). These include CellML, SBML, and SED-ML (Waltemath et al., 2011), which have distinct core objectives and domains of application. Although LEMS was initially inspired by the need for flexible model development in neuroscience it can define a wide range of physio-chemical models, including many in systems biology. The core concepts that distinguish LEMS from the existing model specification systems in systems biology are the use of physical relationships to guide model structure and the ability to express models in compact, low entropy, nested hierarchical forms. LEMS also enforces a strict separation between equations, which belong in the dynamics definition of a *ComponentType*, and parameter values for a specific model which belong in a *Component*. As a generic language for describing hybrid dynamical systems (Goebel et al., 2009), LEMS can therefore be used for developing compact representations of models in systems biology and possibly further afield. Conversion of a circadian rhythm model that includes a gene regulatory network from SBML to LEMS enabled us to run the simulation on both neuroscience specific and generic simulators (Figure 8). We have also been able to pass a significant portion of the SBML Test Suite. These encouraging preliminary results with SBML show that LEMS can act as a bridge between computational neuroscience and systems biology. This raises the exciting prospect of exchanging models and model components across these separate domains and an acceleration in the development of more sophisticated multi-scale neuronal models.

### 3.3. Entropy of model specifications and executable code

A key objective in developing LEMS, was to produce compact, low redundancy standardized representations of the models, hence the choice of the name. However, when models are converted into executable code, most of the structure is removed: dimensional quantities are divided by units to provide dimensionless numbers and mechanistic concepts are converted into a collection of state variables and equations. Ultimately, the numerical code implements state update rules, which correspond to the high entropy end of the spectrum. While it is possible to automate the process of turning a low-entropy representation into a high entropy executable code as used by different simulators, in general it is much more difficult to produce a low entropy representation from a higher entropy one. This will make it difficult for simulators with high entropy model representations to write LEMS directly, because information relating to classes of object are not contained in the simulator representation. However, this can be overcome by adding back this knowledge through manual curation. In the longer term, this problem should be alleviated, as more models are written in LEMS/NeuroML and simulators increasingly support the creation and modification of models in low entropy forms.

### 3.4. LEMS based code generation

A somewhat unexpected outcome of migrating knowledge of the model structure, dimensions and dynamics to LEMS, is the ability to execute models directly and relatively efficiently through the two LEMS interpreters, jLEMS and PyLEMS. We had anticipated that these interpreters would be primarily of interest for model testing, validation, providing reference data, and operating on the LEMS data model for mapping models to other systems. For running realistic scale simulations we had assumed we would need to export models to other tools. However, preliminary work on model flattening and code generation suggests that they may provide a relatively efficient solution for at least some classes of models. To explore further the idea of “simulator-free” model execution via automatic code generation, we implemented template-based exporters, which can generate de novo C and MATLAB code (Figure 7). The models can then be simulated independently from the LEMS interpreters, directly as binary code (in the C case) or from within MATLAB. By delegating low-level numerical computations to standard/built-in libraries in these systems, we obtained very robust and accurate results. The prospect of automated code generation blurs the distinction between code generation and simulators (Figure 10C).

Simulation of more complex models—such as those involving synaptically connected networks of neurons—is not currently possible with our simulator independent automated code generation approach, as the infrastructure for passing spiking events between cells needs to be build from scratch in most neuroscience agnostic target formats. However, we were able to automatically generate code from a LEMS-based network model for NEURON, a domain specific simulator that does support the concept of message passing networks. Although preliminary, this type of code generation directly from the LEMS specification radically alters the potential relationship between model specification and simulation platforms. The possibility of automatically generating code tailored to specific hardware from LEMS could be particularly useful given the growing diversity of hardware for running large models, including GPUs (Brette and Goodman, 2012), FPGAs (Cheung et al., 2012) and novel large parallel systems such as SpiNNaker (Sharp et al., 2012) thereby providing an efficient way to exploit a range of novel hardware that could lower the cost and increase the speed of large scale simulations in computational biology.

### 3.5. Current limitations and future directions

The illustrative examples we have presented show that the current stable release of LEMS and NeuroML 2 provide a flexible and extensive framework for describing models of brain function. Indeed, the LEMS/NeuroML 2 framework has already been used to define the cellular and synaptic properties of a published model of the cerebellar input layer network (Billings et al., 2014). However, several aspects of the framework are still in active development.

While multicompartmental cells can be defined using segments based on LEMS *ComponentType*s, and 3D spatial location can be defined in cartesian coordinates, the language is currently lacking a more sophisticated definition of space. However, a proposal for a new set of geometric primitives is at an advanced stage of development, and this will enable LEMS to represent three dimensional volumes and the reduction of certain classes of PDEs over those volumes to one dimensional PDEs over their axial skeletons. Our initial focus will be on an in-built diffusion operator, that can be used to express both the membrane potential equations and biochemical processes such as calcium diffusion. We expect that this will open up a range of neuroscientifically relevant models without calling for intractable solutions to generic 3D PDEs. Besides the conventional cable equation, this will cover models involving internal reaction-diffusion systems and will include the possibility of expressing external conditions as required, for example, to model extracellular field potentials.

Current limitations to the scope of LEMS that will be more straightforward to address are the representation of noise, implementation of gap junctions and ways to refer to external files for driving inputs or setting initial conditions. Although LEMS provides a *random*() function, more complex noise models would presently have to be implemented with custom *ComponentType*s, which is inefficient. A better solution to this problem would be to utilize a core set of optimized algorithms for generating noise signals with specific properties. A preliminary implementation of electrical synapses has recently been developed (which will also facilitate specification of graded chemical synapses) and will be released in the next version of NeuroML 2. Lastly, there are a number of existing ways for representing data defined in external files that could be used for LEMS (including proposed extensions to the SED-ML specification), and this limitation should be relatively straightforward to resolve.

### 3.6. Reproducibility, accessibility, portability, and transparency of models in computational neuroscience

Computational models are increasingly being used to understand brain function. However, the value of data driven and biophysically detailed modeling as a scientific tool has been diminished by the fact that many published models cannot be easily reproduced or critically evaluated by most neuroscientists. Increasing the Reproducibility, Accessibility, Portability and Transparency (RAPT) of models is therefore an essential prerequisite for them to be more widely adopted by the community as valuable scientific tools. Standardized model descriptions that are machine readable hold the key to this.

By including all of the knowledge required to specify a model, LEMS/NeuroML 2 provides a powerful tool for increasing RAPT. Simulations described in research papers can be specified in LEMS/NeuroML 2 file and reproduced using a compliant simulator or interpreter, subject to numerical approximations and truncation errors. The contents of models specified in LEMS/NeuroML2 can also be parsed and converted into other languages, increasing portability and reuse, or into a more human friendly form. This feature of LEMS/NeuroML 2 is being used by the Open Source Brain initiative^6^, a repository of neuroscience models in standardized format, and in the OpenWorm project^7^. These initiatives show that defining models in LEMS/NeuroML 2 can make their properties accessible and transparent to all interested parties, irrespective of their ability to read code.

Critical evaluation of biologically detailed computational models and feedback from both computational and experimental neuroscientists will be invaluable in improving the biological validity and robustness of models over time. This will be increasingly important as advances in the connectomics field (Helmstaedter et al., 2013) and data produced by large scale brain initiatives (Kandel et al., 2013) lead to the possibility of ever more detailed *in silico* reconstructions of neuronal circuits. By increasing the RAPT of models, LEMS/NeuroML 2 provides a route to improve the scientific value of detailed computational models in neuroscience and beyond.

## 4. Materials and methods

The LEMS language was developed in parallel to the initial reference implementation jLEMS (source code at https://github.com/LEMS/jLEMS), and the serialization of LEMS in XML followed closely the internal classes used in jLEMS for specifying the model. Documentation of all of the elements in LEMS can be found here: http://lems.github.io/LEMS/elements.html. This is automatically generated from documentation in the jLEMS source code. An XML Schema has been developed which can be used to check that an XML file is valid LEMS (the latest version of this can be found on https://github.com/LEMS/LEMS). All of the NeuroML 2 *ComponentType* definition files (see below) are valid against this. However, both jLEMS and PyLEMS allow more flexibility in LEMS files; LEMS elements can be in any order in a file as long as the overall containment rules of the language (Figure 2A) are followed.

The main repository for development of NeuroML 2 is https://github.com/NeuroML/NeuroML2 and there are regular releases of stable verisons of the specification. The version described in this document is the NeuroML 2 beta3 release. XML Schemas for each release are available in the above mentioned repository, and can be used to validate standalone NeuroML files. This Schema can be used to develop NeuroML support without use of the LEMS *ComponentType* definitions. The files containing these *ComponentType* definitions (e.g., Cells.xml, Synapses.xml) can be found in the NeuroML2CoreTypes folder of this repository.

While jLEMS can be used to simulate any valid LEMS model, we have developed a number of other neuroscience specific Java packages to build on this (all of these are available in https://github.com/NeuroML): a full application programming interface (API) in Java for NeuroML 2 (org.neuroml.model); options for exporting LEMS/NeuroML 2 to other formats (e.g., those in Figure 7, org.neuroml.export); options for importing other formats to LEMS (org.neuroml.import); and a single package (jNeuroML) which can be used to bundle all of these into a single Java jar file, along with a utility *jnml* for accessing all this functionality via the command line (e.g., Figure 8). These Java packages are built using Maven (http://maven.apache.org), which facilitates the management of dependencies between different packages. The NeuroML 2 *ComponentType* definitions are included with jNeuroML, allowing execution of NeuroML files with *jnml* (information about the simulation needs to be in a LEMS file which contains a *Simulation* element and imports the NeuroML files). A Python API for reading, writing and validating NeuroML 2 has also been developed (https://github.com/NeuralEnsemble/libNeuroML, Vella et al., 2014).

PyLEMS is a pure Python implementation of the LEMS language, and this can be obtained from: https://github.com/LEMS/pylems. This is a standalone package which can both parse and simulate existing LEMS models and provides an API in Python for reading, modifying and writing LEMS files (Vella et al., 2014). It can also simulate NeuroML 2 models by including the NeuroML 2 *ComponentType* definitions. PyLEMS is also on the Python Package Index, allowing it to be installed using the *setuptools* command *easy_install*.

Supplementary Material 1 consists of a zip file containing the NeuroML 2 *ComponentType* definition files together with all of the LEMS/NeuroML 2 examples presented here (in Figures 2–5, 8, 9) along with versions of jLEMS and PyLEMS which can be used to reproduce the figures. These materials are also available at https://github.com/NeuroML/NML2_LEMS_Examples.

### Conflict of interest statement

The authors declare that the research was conducted in the absence of any commercial or financial relationships that could be construed as a potential conflict of interest.

## Footnotes

^1^http://www.neuroml.org/tool_support.php

^2^http://lems.github.io/LEMS/elements.html

^3^https://github.com/NeuroML/jNeuroML

^4^https://www.modelica.org

^5^http://sbml.org/Software/SBML_Test_Suite

^6^http://www.opensourcebrain.org

^7^http://www.openworm.org
